# Frailty in Traumatic Brain Injury—The Significance of Temporal Muscle Thickness

**DOI:** 10.3390/jcm12247625

**Published:** 2023-12-11

**Authors:** Daniel Dubinski, Sae-Yeon Won, Jonas Meyer-Wilmes, Svorad Trnovec, Artem Rafaelian, Bedjan Behmanesh, Daniel Cantré, Peter Baumgarten, Nazife Dinc, Juergen Konczalla, Matthias Wittstock, Joshua D. Bernstock, Thomas M. Freiman, Florian Gessler

**Affiliations:** 1 Department of Neurosurgery, University Medicine Rostock, 18057 Rostock, Germany; sae-yeon.won@med.uni-rostock.de (S.-Y.W.); j.meyer-wilmes@uni-rostock.de (J.M.-W.); svorad.trnovec@med.uni-rostock.de (S.T.); aarafaelyan@gmail.com (A.R.); bedjan.behmanesh@med.uni-rostock.de (B.B.); thomas.freiman@med.uni-rostock.de (T.M.F.); florian.gessler@med.uni-rostock.de (F.G.); 2 Institute of Diagnostic and Interventional Radiology, Pediatric Radiology and Neuroradiology, University Medicine Rostock, 18057 Rostock, Germany; daniel.cantre@med.uni-rostock.de; 3 Department of Neurosurgery, University Hospital, Schiller University Jena, 07747 Jena, Germany; peter.baumgarten@med.uni-jena.de (P.B.); nazife.dinc@med.uni-jena.de (N.D.); 4 Department of Neurosurgery, Goethe-University Hospital, 60596 Frankfurt am Main, Germany; j.konczalla@med.uni-frankfurt.de; 5 Department of Neurology, University Medicine Rostock, 18057 Rostock, Germany; matthias.wittstock@med.uni-rostock.de; 6 Department of Neurosurgery, Brigham and Women’s Hospital, Harvard Medical School, Boston, MA 02115, USA; jbernstock@bwh.harvard.edu

**Keywords:** traumatic brain injury (TBI), frailty, temporal muscle thickness (TMT), sarcopenia, outcome, risk factors

## Abstract

Background: Temporal muscle thickness (TMT) on cranial CT scans has recently been identified as a prognostic imaging parameter for assessing a patient’s baseline frailty. Here, we analyzed whether TMT correlates with Traumatic brain injury (TBI) severity and whether it can be used to predict outcome(s) after TBI. Methods: We analyzed the radiological and clinical data sets of 193 patients with TBI who were admitted to our institution and correlated the radiological data with clinical outcomes after stratification for TMT. Results: Our analyses showed a significant association between high TMT and increased risk for intracranial hemorrhage (*p* = 0.0135) but improved mRS at 6 months (*p* = 0.001) as compared to patients with low TMT. Congruent with such findings, a lower TMT was associated with falls and reduced outcomes at 6 months (*p* < 0.0001 and *p* < 0.0001). Conclusion: High TMT was robustly associated with head trauma sequelae but was also associated with good clinical outcomes in TBI patients. These findings consolidate the significance of TMT as an objective marker of frailty in TBI patients; such measurements may ultimately be leveraged as prognostic indicators.

## 1. Introduction

It is estimated that ~70 million people worldwide suffer from a traumatic brain injury (TBI) each year [1]; in Europe, TBI contributes to a significant portion of injury-related deaths and the annual incidence of head injury presenting at emergency departments remains extremely high [2]. If patients survive a TBI, they are at an increased risk for the development of cognitive impairments (e.g., attention, memory, arousal and/or concentration deficits), neurodegenerative diseases (e.g., Alzheimer’s and Parkinson’s disease), anxiety and psychosis, substance abuse and personality changes [2]. Several epidemiological studies have established pre-existing mental health conditions, low socioeconomic position, male sex, youth, and prior head trauma as relevant risk factors for TBI [3]. While most of these pre-injury risk factors have been evaluated in detail, there is still a great need to identify the risks factors that influence TBI patients’ outcomes.

Frailty in a medical context refers to a multidimensional syndrome characterized by a decreased physiological reserve and an increased vulnerability to adverse health outcomes [4]. It results from age-associated declines in multiple physiological systems, leading to vulnerability to stressors; the concept of frailty aims to identify individuals who are at higher risk for adverse events like falls, disability and hospitalization, and/or mortality [4,5,6,7].

The potential biological mechanisms underlying the association between TMT and frailty are complex and currently the subject of translational research efforts. On the endocrine and metabolic level, alterations in hormonal levels, including decreased testosterone and growth hormone, may impact muscle protein synthesis and maintenance, potentially affecting TMT and frailty [8,9]. Furthermore, age-related alterations in metabolism can influence muscle protein turnover. The alterations in connective tissues and extracellular matrix components as well as muscle fiber atrophy might impact muscle structure, potentially influencing TMT and contributing to frailty [10].

Due to a decreased capacity to recover from sickness and/or other sequelae, frail people are more likely to experience negative clinical outcomes after injuries. Accordingly, our aim was to determine the association of patients’ TMTs on initial CT scans after TBI as a surrogate parameter for frailty and correlate it with the clinical outcomes.

## 2. Materials and Methods

### 2.1. Patients’ Enrollment and Data Collection

All patients admitted to the neurosurgical department at the University Hospital Rostock between August 2019 and June 2022 with a diagnosis of TBI were eligible to be included in the analysis. Final inclusion criteria were as follows: (1) A clinical diagnosis of TBI that was confirmed on CT scan, and (2) patients >18 years and above. Patient characteristics and medical data were collected via the institution’s electronic database. For this retrospective analysis, ethical approval was obtained from the Ethics Committee of the University Medicine Rostock, Germany (Identification number: A 2021-0112); as a non-interventional monocentric, retrospective study, patient consent was waived. Exclusion criteria included lack of radiological data or hospital discharge <24 h after admission. Data collected included: age at admission, sex, pre-existing conditions, anticoagulation status, course of injury, concomitant injury, median TMT, neurosurgical intervention, clinical course, mRS status at discharge and at 6 months post-TBI.

### 2.2. Image Analysis

**Figure 1:**
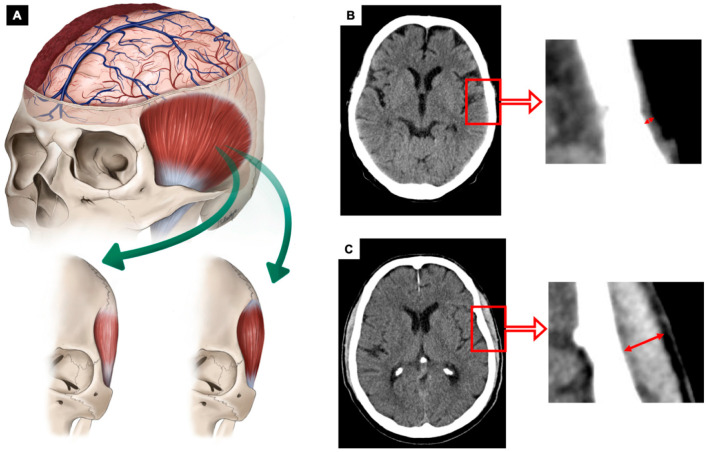
(**A**): Illustration of TBI patients with a right-sided acute subdural hematoma dichotomized according to temporalis muscle thickness (TMT) Green arrows display the dichotomization according to TMT thickness. (**B**,**C**): Representative cases for the assessment of TMT on cranial CT scan shown in red arrows. (**B**): Patient with a low TMT on axial CT scan analysis (bilateral median TMT = 1.5 mm). (**C**): TMT measurement on axial images of a patient with high TMT (bilateral median TMT= 10.5 mm).

Post-traumatic, postoperative (if neurosurgical intervention was performed) and follow-up CT scans were analyzed with PACS software Jivex**^®^** v5.2 (VISUS Technology Transfer GmbH, Bochum, Germany). Image analysis was performed by two neurosurgeons (D.D. and F.G.) who were blinded to patients’ medical data; the plane with the thickest diameter of TMT was used. A representative analysis is displayed in Figure 1. Measurements were carried out on axial CT slices (X, Ymm slice thickness) reconstructed in a standard soft tissue kernel and in standardized soft tissue window setting of (W/L). The temporal muscle was fully visualized bilaterally in a horizontal orientation in the native CT, 5 mm above the superior wall of the orbit. TMT was measured on the left and on the right side separately in each patient and measurements were then added up and divided by two, resulting in a mean TMT per patient. “High” TMT was defined as mean 6–9 mm and “low” TMT as 1–5 mm, as in previous studies:

### 2.3. Study Design

The present analysis is of a retrospective, single-center study assessing the TMT in patients with TBI. Using the initial CT scans after admission for TBI, the aim was (1) to correlate the TMT with clinical course and outcome (2).

### 2.4. Statistics

Data analysis was performed with IBM SPSS Statistics Version 23.0 (SPSS Inc., IBM Corp., Armonk, NY, USA). For patient characteristics, descriptive statistics were used. Fisher’s exact test was used for the comparison of categorical variables between cohorts. For continuous parameters, the Wilcoxon/Mann–Whitney test was used. To assess the impact of the variables, odds ratios (ORs) with 95% confidence intervals (CIs) were calculated. Results with *p* ≤ 0.05 were deemed statistically significant.

## 3. Results

### Participants and Descriptive Analysis

A total of 193 TBI patients were analyzed; the average age was 69 years old (IQR: 52–82) and 123 (64%) of the patients were male. In terms of pre-existing conditions, 93 patients presented with a history of hypertension (48%), 43 with diabetes mellitus (22%), 23 with an oncological disease (12%), 37 with a history of alcohol abuse (19%) and 38 with a history of tobacco abuse (20%). A total of 89 patients (46%) received anticoagulation at admission. See Table 1.

The causes of injury were falls in 136 cases (70%), traffic accidents in 40 cases (21%) and others in 17 patients (9%). Concomitant injuries in addition to TBI were as follows: facial injury was present in 73 patients (38%), thoracic injuries in 28 (15%), abdominal injuries in 5 (3%), skull fractures in 108 (56%), spine injuries in 67 (35%) and intracranial hemorrhage in 88 (46%) patients. The median temporal muscle at admission was 4.75 mm (IQR: 3.65–5.9). The median GCS at discharge was 15 (IQR 14–15) and mRS 2 (IQR 1–4). The median mRS at 6 months was 2 (IQR 1–4).

Characteristics and pre-existing conditions in TBI patients stratified according to temporal muscle thickness.

The univariate analysis showed no significant association between patients’ sex and TMT (*p* = 5.83). A significant association between low TMT and increased age was, however, observed (median age of 78 in the low volume TMT cohort vs. 57 years in the high TMT cohort; *p* = 0.0001). In the analysis of pre-existing health conditions, hypertension demonstrated a significant association with low TMT (*p* = 0.014). Interestingly, tobacco abuse and alcohol abuse were both significantly associated with high TMT (*p* = 0.04 and 0.02). Table 2.

Causes of injury and concomitant injuries stratified by TMT in patients with TBI. In our analysis, falls leading to TBI were significantly associated with decreased TMT (*p* = 0.0007), while traffic accidents were significantly associated with increased TMT (*p* = 0.034). In terms of concomitant injuries, the presence of intracranial hemorrhage was significantly associated with high TMT (*p* = 0.009).

**Figure 2:**
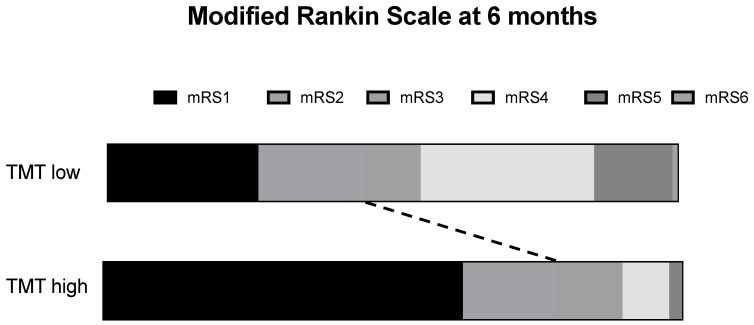
mRS at 6 months stratified into low vs. high temporal muscle thickness. The dashed line indicates the significance difference in good outcome of high TMT patients at six months after TBI. Abbreviations: TMT: temporal muscle thickness; mRS: modified Rankin scale.

The outcomes were stratified by temporal muscle thickness in TBI patients. In terms of mRS at discharge, we found a significant association between a good outcome and high TMT (*p* = 0.0001). This finding was continued at 6 months post-TBI with a median mRS of 1 in the high TMT cohort vs. 2 in the low TMT cohort (*p* = 0.001). See Table 2 and Figure 2.

## 4. Discussion

This study investigated the value of TMT as derived from cranial CT scans preformed on patients who were admitted for management for TBIs. The major findings relate to the significant association of low TMT and reduced outcome status at 6 months after TBI. In line with such findings, high TMT in TBI patients was significantly associated with increased rates of associated intracranial hemorrhage yet at the same time better functional outcomes on the mRS at discharge and at 6 months. This finding highlights the relevance of TMT as an objective factor with prognostic value, novel for TBI patients.

In our cohort, low TMT was significantly associated with increased age, a population in which the management of TBI can be challenging due to reduced homeostatic reserves in response to trauma [11,12,13]. On one hand, elderly people aged ≥75 years reportedly lose skeletal muscle mass at an annual rate of approximately 1% [14]. On the other hand, however, seeing the association between TMT and age as the main reason for reduced clinical outcomes would be wrong as a linear relationship between TMT and age is not ubiquitously verified and there is certainly a cohort of older patients without sarcopenia [15,16].

Older adults are also increasingly likely to receive anticoagulant therapy, which increases susceptibility to bleeding and may increase injury severity. Interestingly, although in our cohort reduced TMT was associated with anticoagulation therapy, the risk for intracranial bleeding was significantly reduced. This finding is of interest and could at least in part be explained by the fact that younger age is associated with increased risk-taking behavior which may increase injury risk in general and that of intracranial hemorrhaging in TBI in specific [17,18]. Our finding of the significant association between high TMT and drug intake (tobacco and alcohol) is therefore in line with the known literature and emphasizes the representability of our cohort [17]. The significant association of falls as the main etiology for TBI in sarcopenic (low TMT) patients’ is also in line with the current literature where older adults with TBI over 50% are reported to be prone to falls [1,19]. The association of low TMT and hypertension is also notable: chronic hypertension can lead to arterial stiffness and reduced blood flow to tissues, potentially impacting muscle function. Furthermore, impaired microcirculation due to hypertension may affect nutrient and oxygen delivery to muscles, influencing their structure and possibly contributing to changes in TMT.

Having summarized until now, a high TMT shows an association with young age, high-velocity trauma (traffic accident) and intracranial hemorrhage, but still leads to better outcomes. Frail TBI patients with reduced TMT on the other hand were more prone to poor outcomes despite a reduced association with intracranial hemorrhage. This initially paradoxical fact may be rationalized as follows: patient frailty outweighs even the increased TBI sequelae seen in younger patients with high TMT due to a lowered recovery capacity.

Due to the demographic change and the continuously aging population, TBIs of the elderly will play an increasingly important role in neurosurgery and acute care and reliable and easy to obtain risk factors are and will be of great clinical use [1]. However, the acute care of elderly TBI patients is frequently assumed to be futile and some facilities have even age restrictions that prevent them from admitting older TBI patients to neuro-intensive care or neurosurgical intervention [20]. Our finding challenges this old concept as older TBI patients with high TMT may recover effectively, indicating that TBI severity and chronological age alone are insufficient prognostic indicators.

While our analysis demonstrates the value of TMT in a sizable cohort of TBI patients, our study faces some limitations. The retrospective analysis of TMT prohibited the evaluation of anatomical-functional relationships. Furthermore, as this was a retrospective observational study, confounding, selection bias, and uncontrolled statistical error risk cannot be excluded. Hence, further prospective randomized trials with large cohorts are necessary to validate our findings.

## 5. Conclusions

Patients with high TMT showed good TBI recovery rates, enabling its use for prognostication after TBI in the future and challenging thereby old established risk factors such as chronological age and bleeding patterns.

## Figures and Tables

**Table 1:** Demographics and outcome data. Abbreviations: IQR: Interquartile range, GCS: Glasgow Coma Scale, mRS: Modified Rankin Scale.

Patient Characteristic	(n = 193)
**Male**, n (%)	123 (64)
**Age**, median (IQR)	69 (52–82)
**Pre-existing conditions**	
Hypertension, n (%)	93 (48)
Diabetes mellitus, n (%)	43 (22)
Oncological disease, n (%)	24 (12)
Alcohol abuse, n (%)	37 (19)
Tobacco abuse, n (%)	38 (20)
Anticoagulation, n (%)	89 (46)
Antiplatelet therapy, n (%)	52 (27)
**Cause of injury**, n (%)	
Fall	136 (70)
Traffic accident	40 (21)
Other	17 (9)
**Concomitant injury**, n (%)	
Face	73 (38)
Thorax	28 (15)
Abdomen	5 (3)
Extremity	46 (24)
Skull	108 (56)
Spine	67 (35)
Cerebral hemorrhage	88 (46)
**Radiological parameters**	
TMT mean median, mm (IQR)	4.75 (3.65–5.9)
**Status at discharge**	
GCS at discharge, median (IQR)	15 (15–15)
mRS at discharge, median (IQR)	2 (1–4)
**Outcome**	
mRS 6 months, median (IQR)	2 (1–4)

**Table 2:** Univariate analysis of juxtaposed characteristics according to TMT in TBI patients. Abbreviations: OR: odds ratio, IQR: Interquartile range, GCS: Glasgow Coma Scale, mRS: Modified Rankin Scale.

Patient Characteristic	Low TMT	High TMT		Univariate	
n = 193	n = 104	n = 89	OR	95% CI	*p*-Value
**Sex**					
male, n (%)	47 (45)	76 (85)	0.14	0.07–0.29	5.832
**Age**, median (IQR)	78 (65–85)	57 (39–69)	-	11.5–22.46	0.0001
**Pre-existing comorbidities**					
Hypertension, n (%)	59 (57)	34 (38)	2.1	1.19–3.78	0.014
Diabetes mellitus, n (%)	28 (27)	15 (17)	1.8	0.90–3.68	0.118
Oncological disease, n (%)	15 (14)	9 (10)	1.5	0.62–3.61	0.391
Alcohol abuse, n (%)	14 (13)	23 (26)	0.4	0.21–0.93	0.043
Tobacco abuse, n (%)	14 (13)	24 (27)	0.4	0.20–0.88	0.028
Anticoagulation, n (%)	51 (49)	38 (43)	1.3	0.73–2.28	0.389
Antiplatelet therapy, n (%)	33 (32)	19 (21)	1.7	0.89–3.29	0.143
**Cause of injury**					
Fall, n (%)	86 (83)	50 (56)	3.7	1.93–7.20	0.00007
Traffic accident, n (%)	15 (15)	25 (27)	0.5	0.24–1.00	0.037
Other, n (%)	2 (2)	15 (17)	0.1	0.02–0.43	0.002
**Concomitant injury**					
Face, n (%)	37 (36)	36 (40)	0.8	0.45–1.46	0.551
Thorax, n (%)	15 (14)	13 (15)	1	0.44–2.22	1
Abdomen, n (%)	2 (2)	3 (3)	0.6	0.09–3.44	0.663
Extremity, n (%)	23 (22)	23 (26)	0.8	0.42–1.58	0.612
Skull, n (%)	57 (55)	51 (57)	0.9	0.51–1.62	0.772
Spine, n (%)	37 (36)	30 (34)	1.1	0.61–1.97	0.879
Cerebral hemorrhage, n (%)	55 (53)	33 (37)	1.9	1.07–3.93	0.031
**Status at discharge**					
GCS at discharge,median (IQR)	15 (14–15)	15 (15–15)	-	0.19–0.53	0.371
mRS at discharge,median (IQR)	3 (1–4)	1 (1–2)	-	0.79–1.54	0.0001
**Outcome**					
mRS 6 months, median (IQR)	2 (1–4)	1 (1–4)	-	0.65–1.34	0.001

## Data Availability

The data presented in this study are available on request from the corresponding author.
